# Unusual Age of Presentation and Etiology of Slipped Capital Femoral Epiphysis Following a Seizure Attack: A Case Report

**DOI:** 10.7759/cureus.30772

**Published:** 2022-10-27

**Authors:** Ahmed H Kaneetah, Majed N Alosaimi, Ahmed A Ismail, Ahmad O Alansari

**Affiliations:** 1 Orthopedic Surgery, King Abdullah International Medical Research Center, Jeddah, SAU

**Keywords:** pediatric seizure, trauma pediatric, pediatric orthopedic surgery, dandy-walker, slipped femoral epiphysis

## Abstract

Slipped capital femoral epiphysis (SCFE) is a common adolescent hip disorder affecting adolescents between eight and 15 years of age. Therefore, few studies in the literature address children under the age of 10 years with SCFE. Obesity is a well-known predisposing factor for SCFE. Increased body mass index, in addition to high activity levels, may cause shearing forces during normal activities that may cause a slip in children less than 10 years of age. This paper reports a rare case of SCFE in a 40-month-old girl with an unusual etiology of a seizure attack. Awareness regarding this condition by observing the presentation of symptoms and radiographic findings is useful to rthopedic surgeons in its management.

## Introduction

Slipped capital femoral epiphysis (SCFE) is the posterior and medial slippage of the capital femoral epiphysis on the femoral neck. It is one of the most common adolescent hip disorders. Boys are affected more frequently than girls [1-4]. In most cases, the etiology is unknown but thought to be multifactorial, including risk factors of obesity and endocrine disorders such as hypothyroidism, growth hormone supplementation, hypogonadism, renal osteodystrophy, and panhypopituitarism [1, 5-9]. Patients usually present with persistent hip, groin, thigh, or knee pain that is poorly localized and walk with an antalgic gait [10,11]. On clinical examination, pain is elicited on passive motion of the hip, and a loss of range of motion in internal rotation and abduction is noted. When the hip is flexed, the lower extremity often rotates externally as a result of the orientation of the capital epiphysis on the femoral neck [10,12,13]. It is classified as stable or unstable based on the stability of the physis. In stable SCFE, patients can walk without walking aids, and in unstable SCFE, patients cannot walk even with the use of walking aids [14,15]. Diagnosis is usually made with anteroposterior and lateral (frog-leg views in patients with stable SCFE and cross-table lateral views in unstable SCFE) radiographs of both hips [16]. Management is usually operative with percutaneous in situ fixation. As mentioned earlier, SCFE usually affects adolescents and is usually seen in boys between 12 and 15 years of age or girls between 10 and 13 years of age. In this article, we present an unusual age presentation and etiology of SCFE.

## Case presentation

**Figure 1:**
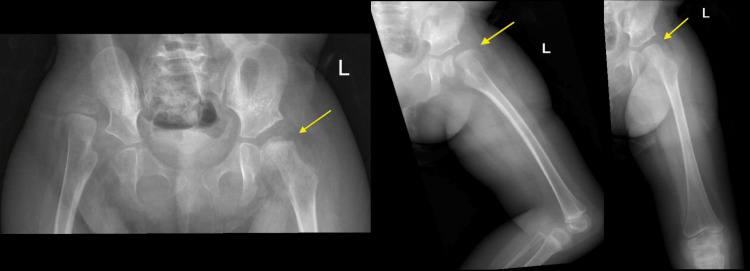
The X-ray of the patient's left femur at the time of injury

Our patient is a 40-month-old girl, with a known case of seizures, sensorineural deafness, ataxia, mental retardation, and electrolyte imbalance (SeSAME) syndrome, Dandy-Walker syndrome, hypotonia, and generalized developmental delay. Her code status is comfort care by general pediatrics. Perinatal history is as follows: full-term baby, a product of cesarean section due to failure to progress with no complications during pregnancy. The patient had a history of multiple admissions to the neonatal intensive care unit (NICU) and regular wards due to seizure disorder. She is not a walker and cannot sit unassisted. She was referred to orthopedics when she was two years of age for right hip dislocation and left shoulder recurrent dislocations managed with closed reduction and spica casting followed by abduction brace but remained subluxed; shoulder closed reduction was done but with many subsequent dislocations/relocations. She presented to the emergency department after a seizure attack. The mother heard a clicking sound over the left hip and noticed her baby was uncomfortable and complaining of pain. In the preceding three days before the presentation, she had multiple seizure attacks around three times per day, lasting for seconds to minutes, then stopping. Prior to that, there were no previous attacks in the last three months. She had a history of subjective fever at home; however, no clear focus of infection was identified. She was referred from the emergency department to orthopedics as a case of a left hip fracture and referred to general pediatrics for seizure management. Upon examination in the emergency department, she looked unwell but not toxic, and she was in pain. She was vitally stable and afebrile at that time. Her left lower limb was abducted, semi-flexed, and in an external rotation position. There was mild swelling over the left proximal thigh but no wounds or bruises. There was tenderness isolated over the proximal thigh and left hip. Back, knee, and ankle examinations revealed no tenderness. She was moving her foot and her pulse was palpable. A head-to-toe examination was carried out and was insignificant for any other recent injury or trauma. The X-rays of the left femur showed slipped epiphysis to the medial inferior aspect and the left femoral metaphysis displaced to the superior lateral aspect (Figure 1). The X-rays of the lumbar spine and ipsilateral knee were unremarkable.

**Figure 2:**
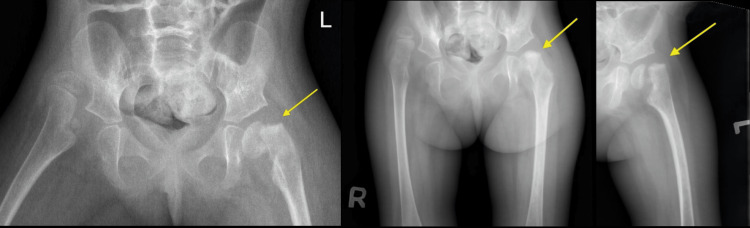
The X-ray of the patient taken six weeks post-injury

**Figure 3:**
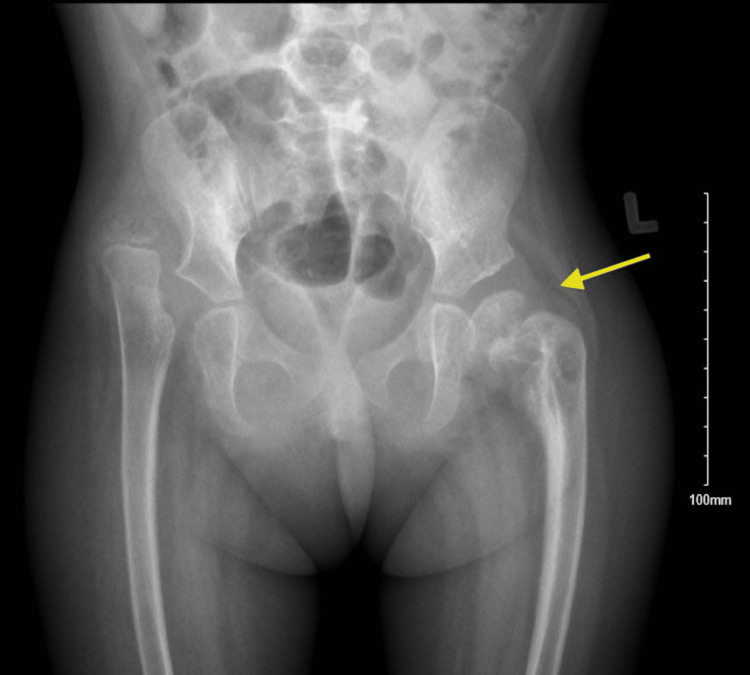
The X-ray of the patient taken 12 months post-injury

Good pain medications were given to the patient, and she was put on a plaster of Paris slab from the ankle to the buttock area, as the hip spica was not safe since the patient had multiple seizures in the previous few days. She was admitted for almost two weeks by general pediatrics for seizure control. She was followed up later in the clinic; after two weeks, the slab was changed as it was ruined in the proximal part due to cleaning issues. She was followed up after four weeks, six weeks, eight weeks, three months, and one year after the injury. The slab was removed in around six weeks. The patient was looking fine with no pain or tenderness over the hip with a good passive range of motion comparable to the pre-injury level, as per the family. The X-rays showed a healed slipped capital femoral head epiphysis in its position (Figure 2). Over the next 12 months, the patient was followed up in the clinic with no new active issues. The last X-ray was taken 12 months post-injury (Figure 3). 

## Discussion

Slipped capital femoral epiphysis is a common adolescent hip disorder affecting adolescents between eight and 15 years of age. [1] Few case reports and case series exist in the literature that addresses children under the age of 10 with SCFE [17-20]. Azzopardi et al. presented a case series of 10 children with SCFE with an age ranging from 5.2 to 9.9 years [19]. Another case series by Chatziravdeli et al. had nine patients with SCFE where the mean age at presentation was 8.25 years, and the youngest was six years of age [17]. Al Qahtani et al. presented a case of idiopathic bilateral SCFE in a five-year-old boy who underwent staged bilateral pinning in situ [20]. To the best of our knowledge, only one case has been reported in the literature up to date with a patient less than four years old diagnosed with SCFE. This patient was 10 months of age, known to have epileptic encephalopathy, and developed SCFE in the left hip after a seizure attack. He was managed with closed reduction and a hip spica cast application [21]. Our patient was 40 months (3.33 years) of age when diagnosed with SCFE after a seizure attack. Obesity is a well-known predisposing factor for SCFE. Increased body mass index, in addition to high activity levels, may cause shearing forces during normal activities that may cause a slip in children less than 10 years of age [7,18, 22-25]. Our patient does not walk or sit unsupported and her BMI at presentation was 13.84 and she is below the fifth centile both in weight and height. The SCFE is classified into stable and unstable based on the stability of the physics and the ability to bear weight. In stable SCFE, the presenting complaint is usually groin pain, which may be referred to as the anteromedial aspect of the thigh and knee, and in some, it may only be localized to the lower thigh or knee. Those patients will have an antalgic limp, with the affected side in an increased external rotation position. On the other hand, patients with unstable SCFE report sudden and severe pain in the affected hip region, usually as the result of a relatively minor fall [1,14,15]. Our patient was classified as unstable SCFE.

The management of SCFE is usually operative with percutaneous in situ fixation as the gold standard treatment. However, in our patient, due to multiple factors, including multiple comorbidities and the code status of the patient, the decision was made to go for non-operative management. Closed reduction was not attempted as she is not a walker and would not benefit much from such a procedure. The hip spica cast was not applied as in the previously mentioned study [21] as the patient was having multiple episodes of seizures and it was not safe to be applied. Instead, a simple slab from the foot to the buttock area was applied and was removed after around six weeks, with the patient returning to almost the same pre-injury status in the subsequent six months after the injury.

## Conclusions

Slipped capital femoral epiphysis is a common hip disorder frequently affecting the adolescent age group. Patients usually present with persistent hip, groin, thigh, or knee pain that is poorly localized and walk with an antalgic gait. Often, the etiology is unknown but thought to be multifactorial with risk factors such as obesity and endocrine disorders such as hypothyroidism, growth hormone supplementation, hypogonadism, and panhypopituitarism. Here, we report a case of SCFE with an uncommon age of presentation and etiology. Although the gold standard of management is operative with percutaneous in situ fixation, the management applied in our case was only immobilization with slab application and good pain control with the coexistence of multiple comorbidities, and showed acceptable outcomes.
